# Supplementary material for: DMRforPairs: identifying Differentially Methylated Regions between unique samples using array based methylation profiles
Source: BMC Bioinformatics. 2014 May 15;15:141. doi: 10.1186/1471-2105-15-141 (PMC4046028; doi:10.1186/1471-2105-15-141)
Supplement: Additional file 2 — DMRforPairs output for the comparison of A431-MCF7 and NA17018-NA17105. Please start from the HTML files in each folder. Available via the BMC Bioinformatics website. [file 1471-2105-15-141-S2.zip › 1394847754114233_MOESM2_ESM/A431_MCF7/figures/1124.pdf]

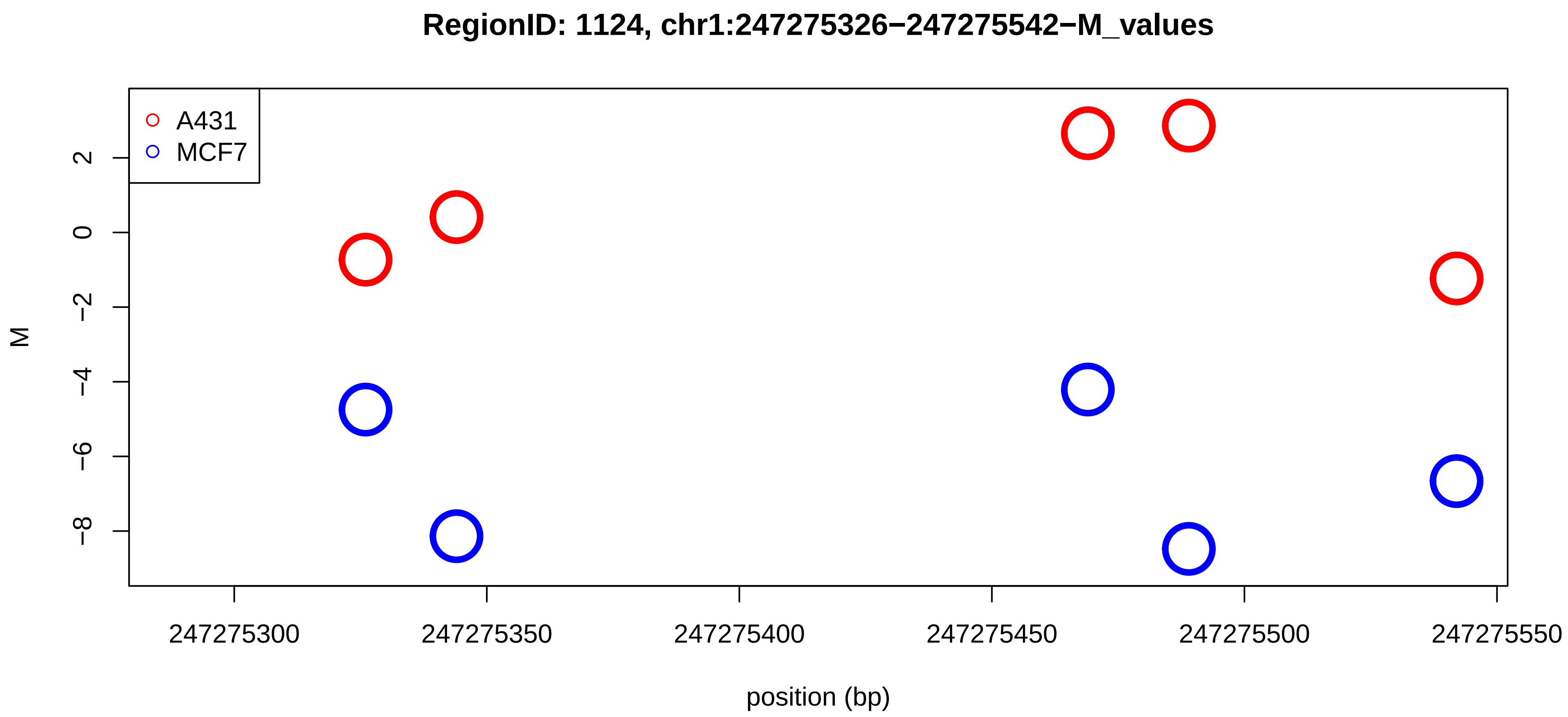

RegionID: 1124, chr1:247275326−247275542−M_values
A431
MCF7
M
2
0
−2
−4
−6
−8
247275300
247275350
247275400
247275450
247275500
247275550
position (bp)

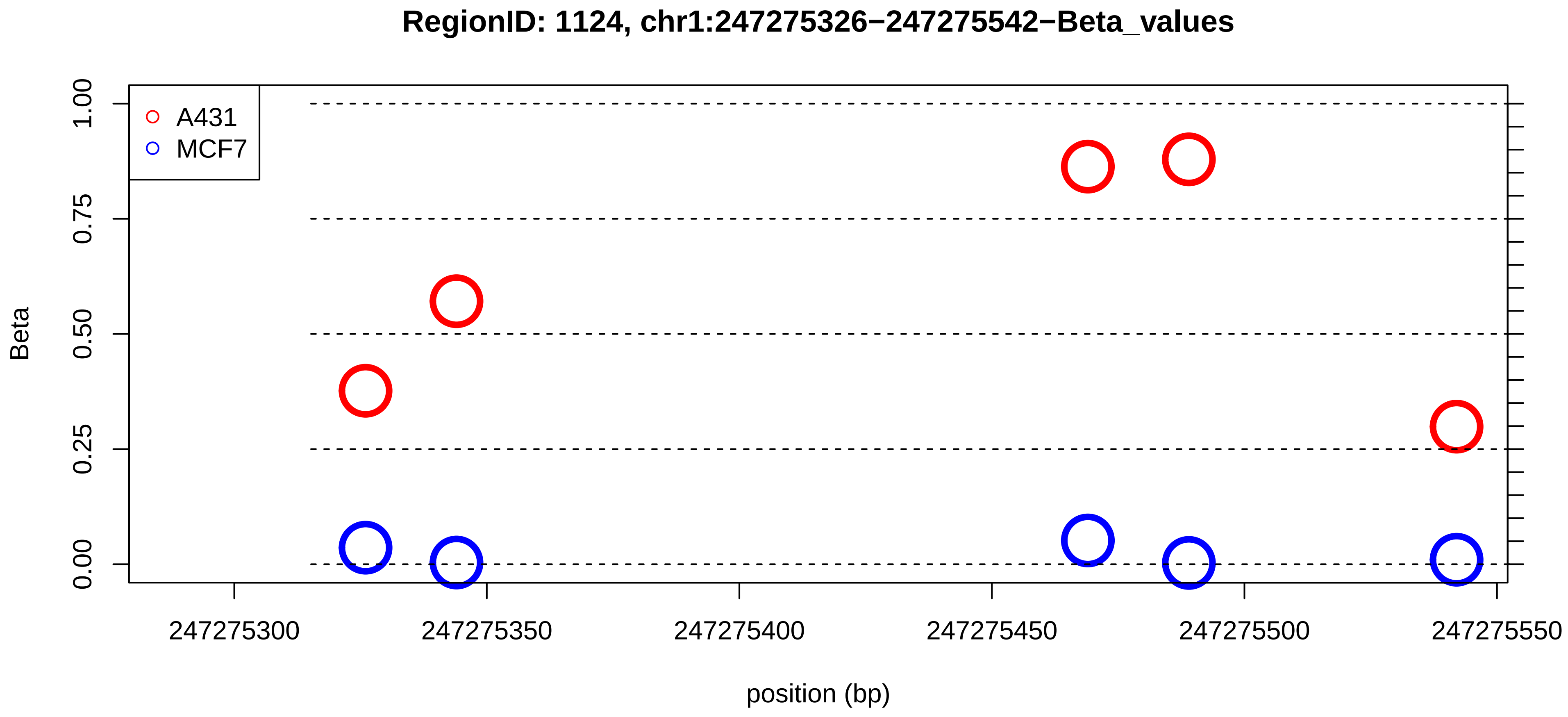

RegionID: 1124, chr1:247275326−247275542−Beta_values
A431
MCF7
Beta
1.00
0.75
0.50
0.25
0.00
247275300
247275350
247275400
247275450
247275500
247275550
position (bp)

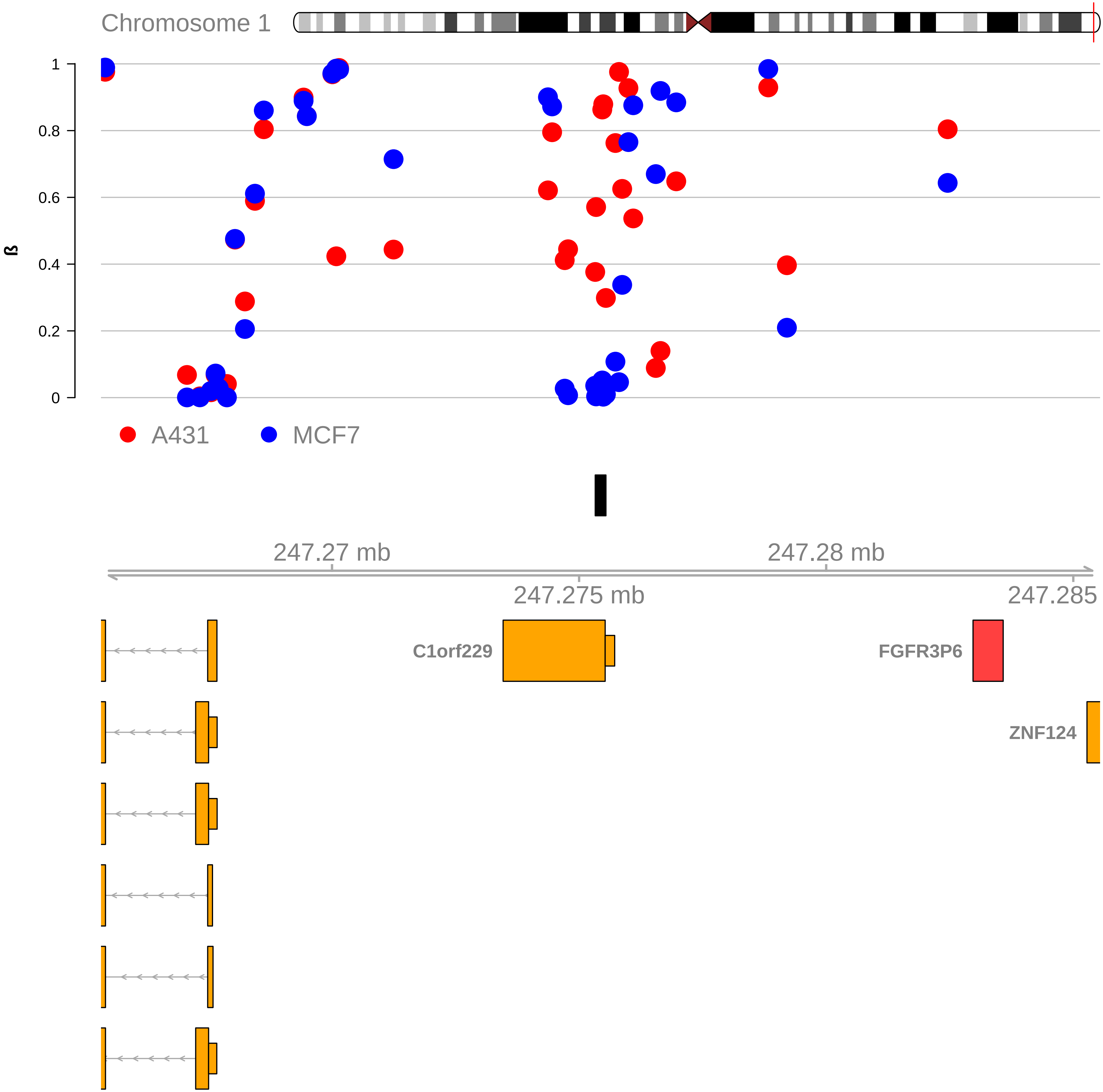

Chromosome 1
1
0.8
0.6
0.4
0.2
0
ß
A431
MCF7
247.27 mb
247.28 mb
247.275 mb
247.285 r
C1orf229
FGFR3P6
ZNF124
